# Application of artificial intelligence in cervical cytology: a systematic review of deep learning models, datasets, and reported metrics

**DOI:** 10.3389/fdata.2025.1678863

**Published:** 2026-01-02

**Authors:** Miguel Angel Valles-Coral, Lloy Pinedo, Ciro Rodríguez, Diego Rodríguez, Keller Sánchez-Dávila, Lolita Arévalo-Fasanando, Nelly Reátegui-Lozano

**Affiliations:** 1 Facultad de Ingeniería de Sistemas e Informática, Universidad Nacional Mayor de San Marcos, Lima, Peru; 2 Facultad de Ingeniería de Sistemas e Informática, Universidad Nacional de San Martín, Tarapoto, Peru; 3 Facultad de Ingeniería y Negocios, Universidad Privada Norbert Wiener, Lima, Peru; 4 Facultad de Ciencias de la Salud, Medicine, Universidad Peruana de Ciencias Aplicadas (UPC), Lima, Peru; 5 Facultad de Medicina Humana, Universidad Nacional de San Martín, Tarapoto, Peru; 6 Facultad de Ciencias de la Salud, Universidad Nacional de San Martín, Tarapoto, Peru

**Keywords:** cervical cytology, cancer, deep learning, models, datasets, metrics

## Abstract

**Introduction:**

The use of artificial intelligence (AI) in cervical cytology has increased substantially due to the need for automated tools that support the early detection of precancerous lesions.

**Methods:**

This systematic review examined deep learning models applied to cervical cytology images, focusing on the architectures used, the datasets employed, and the performance metrics reported. Articles published between 2022 and 2025 were retrieved from Scopus using PRISMA methodology. After applying inclusion criteria and full-text screening, 77 studies were included for RQ1 (models), 75 for RQ2 (datasets), and 71 for RQ3 (metrics).

**Results:**

Hybrid models were the most prevalent (56%), followed by convolutional neural networks (CNNs) and a growing number of Vision Transformer (ViT)-based approaches. SIPaKMeD and Herlev were the most frequently used datasets, although the use of private datasets is increasing. Accuracy was the most commonly reported metric (mean 87.76%), followed by precision, recall, and F1-score. Several hybrid and ViT-based models exceeded 92% accuracy. Identified limitations included limited cross-validation, reduced clinical representativeness of datasets, and inconsistent diagnostic criteria.

**Discussion:**

This review synthesizes current trends in AI-based cervical cytology, highlights common methodological limitations, and proposes directions for future research to enhance clinical applicability and standardization.

## 1 Introduction

Cervical cancer remains one of the leading causes of death among women worldwide, particularly in countries with limited access to healthcare services (Torres-Roman et al., 2021). The Papanicolaou test has, for decades, enabled early detection of cellular abnormalities in the cervix, helping to prevent their progression to invasive cancer (Papanicolaou and Traut, 1941). This review aims to systematically analyze studies that apply artificial intelligence (AI) in cervical cytology, focusing on the models and datasets used, as well as the main performance outcomes.

Computational solutions in medicine have evolved from simple heuristic systems based on rule sets to more complex deep learning models, particularly in medical imaging (Cabral et al., 2025; Bolia and Joshi, 2025). Currently, with reduced computational costs, there is increasing interest in hybrid architectures that combine convolutional neural networks (CNNs) with vision transformer (ViT)-based models, which exhibit superior ability to identify complex patterns in cellular images and aid in cytopathological diagnosis (Maurya et al., 2023; Hong et al., 2024). Recent advances in AI have also demonstrated applications beyond cytology, such as transcriptomic event inference in cancer cells and drug response prediction using graph-based models (Eralp and Sefer, 2024; Sefer, 2025). These developments highlight the broad potential of AI in oncology and reinforce the relevance of its application to cervical cytology. Despite these advances, important limitations remain, such as the flawed assumption that cell classification alone is sufficient for cancer diagnosis, the poor quality and representativeness of the datasets used, and the excessive complexity of some models.

The use of AI in cervical cytology seeks to assist in the automatic identification of suspicious cytological lesions (Tang et al., 2023; Shinde et al., 2022), as part of an automated pipeline for early detection of cellular abnormalities. This involves computer vision models analyzing microscopic images, classifying cells based on morphological features, and diagnosing cellular lesion levels, primarily using supervised learning algorithms (Mohammed et al., 2022; Wang et al., 2024; Cheng et al., 2021). While this automation can facilitate clinical workflows, it also poses risks. A poorly constructed, parametrized, or trained model could produce false positives or negatives, compromising patient care. Moreover, if healthcare providers distrust the model's outputs, they may avoid using it or use it improperly. Trust depends on how well the model's decisions are understood (Martínez, 2005). Thus, good technical performance alone is insufficient; clinical context and other considerations must also be addressed.

Recent years have seen a surge in research applying AI to cytological image analysis (Dhawan et al., 2018; Gorantla et al., 2019; Kavitha et al., 2023), with growing interest in CNNs, ViTs, regression-based models, or their combinations. These studies fall into two main categories: those that rely on public datasets such as Herlev, SIPaKMeD, or Mendeley LBC (Ouh et al., 2024; Chowdary et al., 2023; Chauhan et al., 2023), and those that propose new architectures tailored to specific tasks like detection, segmentation, or classification using proprietary datasets (Cheng et al., 2021; Kanavati et al., 2022). While these contributions demonstrate that certain tasks traditionally performed by cytopathologists can be automated with reasonable accuracy, many of them rely on datasets that do not reflect real clinical contexts. Additionally, a frequent conceptual error is equating the detection of cytological anomalies with cancer diagnosis.

The earliest attempts to automate cervical cytology with deep learning relied heavily on CNN-based architectures applied to small, well-curated datasets such as Herlev or SIPaKMeD. These studies demonstrated that automatic recognition of precancerous lesions was technically feasible, though often limited by overfitting and narrow class diversity (Devi et al., 2023). Refinements soon followed with optimized convolutional pipelines or hybridized CNN–GRU variants that improved sensitivity to complex cytological patterns (Rohini and Kavitha, 2024). Others explored tailored CNN designs for Pap smear images, reporting encouraging accuracies but mostly within closed datasets that lacked external validation (Khozaimi and Mahmudy, 2024).

From 2022 onwards, a new wave of studies emphasized hybrid pipelines that combined deep features with classical classifiers or optimization heuristics. For example, hybridization with fuzzy neural networks or ensemble learning improved robustness against inter-sample variability (Kalbhor et al., 2023b). In parallel, researchers began to incorporate non-traditional datasets, including liquid-based cytology and field-of-view tiles from whole-slide images (Gao et al., 2022). These approaches sought to move beyond isolated single-cell images, capturing contextual information closer to real practice, although issues of transparency and reproducibility persisted.

More recently, the field has witnessed the entrance of transformer-inspired backbones and knowledge distillation frameworks, aiming to capture long-range morphological dependencies and optimize computational costs (Kang and Li, 2024). Studies have also experimented with graph-based models and metaheuristic optimizations to enhance precancerous lesion detection, reporting near-perfect accuracies in benchmark datasets but with uncertain clinical transferability (Song et al., 2024). Taken together, these contributions reflect an energetic but fragmented landscape: while technical metrics frequently surpass 90% accuracy, the lack of dataset diversity, external validation, and standardized reporting highlights the persistent gap between benchmark-driven innovation and real-world clinical needs.

This review examines studies applying AI models to classification and diagnostic tasks in cervical cytology, emphasizing how models were constructed, what data they used, and what metrics were reported. In this context, AI refers to algorithms capable of learning from cytological images to identify cellular patterns associated with potential abnormalities. This field integrates computer vision, deep learning, public health, and cytopathology, aiming to develop practical solutions for the early identification of atypical cellular patterns. Common challenges include models that lack generalizability, data that fail to reflect the complexity of real cytology slides (often composed of isolated, well-selected cell images), and limited use of clinical variables that may impact diagnostic decisions (Ssedyabane et al., 2024; Gafeer et al., 2025).

The number of publications in this area has increased notably, reflecting strong scientific interest. Within this context, the present review offers a comprehensive perspective that not only systematizes deep learning models applied to cervical cytology, but also compares the most widely used datasets and provides a cross-sectional analysis of reported performance metrics (Alias et al., 2022; Allahqoli et al., 2022). Although some studies have recently begun addressing issues such as model explainability (Hemalatha et al., 2023), this remains in early stages. The wide variety of approaches and techniques makes it difficult to compare results and establish standards. There is still a need for a review that not only aggregates studies but also critically analyzes their technical limitations in light of the clinical settings where they might be applied.

A systematic review was conducted using the PRISMA methodology to examine AI applications in cervical cytology. Unlike prior reviews, this study offers a critical perspective, distinguishing between cellular lesion detection and cancer diagnosis, and interrogating the conceptual and ethical foundations of the evaluated models. Articles were retrieved from the Scopus database using well-defined inclusion and exclusion criteria, and the data were structured for both qualitative and quantitative analysis. This review aims to guide future research, enhance existing models, and promote responsible use of AI in clinical contexts. Its main contribution lies in a detailed characterization of the most frequently used architectures, datasets, and performance metrics, complemented by a cross-analysis linking model types, data sources, and diagnostic accuracy. Additionally, the review synthesizes recurring patterns and emerging trends to help guide future studies toward more efficient and clinically applicable AI solutions.

## 2 Methodology

This systematic review aims to identify and analyze studies that apply artificial intelligence (AI) to the classification of cervical cytology images, with special attention to the most commonly used models, the datasets employed, and the performance metrics reported. The review followed a three-phase process: planning, execution, and reporting, aligned with PRISMA guidelines and the PICO strategy. The review process was supported by the use of Mendeley (version 1.19.8) for reference management, Excel for tracking study selection, and draw.io (online version, accessed June 13, 2025) for creating the PRISMA flow diagram.

### 2.1 Review planning

To structure the search strategy and clarify the key terms, the PICO framework was adapted to the context of this review (Table 1).

**Table 1:** Application of the PICO method.

**Criterion**	**Description**
Population (P)	Cervical cytology images.
Intervention (I)	AI models for image classification (CNN, ViT, hybrid models).
Comparison (C)	Comparison across different model architectures, classical vs. emerging datasets, and metrics.
Outcome (O)	Types of models used, datasets applied, reported performance metrics, and current trends.

In this phase, a strategy was designed to identify empirical studies that effectively address the research questions. Table 2 describes the questions guiding this systematic review.

**Table 2:** Research questions and objectives.

**Research question (RQ)**	**Objective**
RQ1: What types of AI models (monolithic, hybrid, or others) have been used in cervical cytology image classification, and what trends are observed in recent approaches?	To identify the most applied AI models in cervical cytology and characterize current trends.
RQ2: What datasets have been most frequently used in studies applying AI to cervical cytology, and what new datasets are emerging?	To determine the most used datasets and describe the characteristics of emerging datasets.
RQ3: What performance metrics are most frequently reported in studies applying AI to cervical cytology image classification, and what are the typical values?	To identify the most common performance metrics and analyze reported values in recent studies.

### 2.2 Search strategy and criteria

The search strategy was defined based on the three research questions posed in the planning phase, with specific search strings developed for each (see Table 3). These queries were executed independently in the Scopus database using the Advanced Search option. For each question, key terms and relevant synonyms were defined to maximize retrieval and ensure reproducibility. Additionally, inclusion and exclusion criteria were established as follows:

**Table 3:** Search strings per research question.

**Key criteria**	**Description**
Search string	**RQ1:** TITLE-ABS-KEY(“artificial intelligence” OR “machine learning” OR “deep learning” OR “convolutional neural network” OR CNN OR “vision transformer” OR ViT OR “transformer-based model”) AND TITLE-ABS-KEY(“cervical cytology” OR “pap smear” OR “cervical cells” OR “cervical cytological images”) AND TITLE-ABS-KEY(“classification” OR “cell classification” OR “lesion classification” OR “lesion detection” OR “automatic diagnosis”) **RQ2:** TITLE-ABS-KEY(“artificial intelligence” OR “machine learning” OR “deep learning” OR “convolutional neural network” OR CNN OR “vision transformer” OR ViT OR “transformer-based model”) AND TITLE-ABS-KEY(“cervical cytology” OR “pap smear” OR “cervical cells” OR “cervical cytological images”) AND TITLE-ABS-KEY(“dataset” OR “database” OR “image collection” OR “image repository” OR “public dataset” OR “private dataset” OR “labeled data”) **RQ3:** TITLE-ABS-KEY(“artificial intelligence” OR “machine learning” OR “deep learning” OR “convolutional neural network” OR CNN OR “vision transformer” OR ViT OR “transformer-based model”) AND TITLE-ABS-KEY(“cervical cytology” OR “pap smear” OR “cervical cells” OR “cervical cytological images”) AND TITLE-ABS-KEY(“classification” OR “cell classification” OR “lesion classification” OR “lesion detection” OR “automatic diagnosis”) AND TITLE-ABS-KEY(“performance metric” OR “accuracy” OR “precision” OR “recall” OR “sensitivity” OR “specificity” OR “F1-score” OR “AUC” OR “ROC curve”)
Intervention criteria	Peer-reviewed journal articles. Full-text available in English or Spanish. Keywords related to AI in cervical cytology, datasets, and performance metrics. Publication date between 2019 and 2024
Exclusion criteria	Articles published before 2022. Studies not using AI models. Clinical studies without computational models. Reviews without new data. Works without full-text access
Search mode	Applied to title, abstract, and keywords.

Each query was designed to independently address one of the review's research questions (RQ1, RQ2, RQ3), ensuring traceability, reproducibility, and alignment with the review's objectives.

### 2.3 Keywords and relation to research questions

Table 4 summarizes the keywords used in the search strategy and their relation to the research questions.

**Table 4:** Keywords, synonyms, and related research questions.

**Keyword/term**	**Synonyms/variants**	**Related to**
Artificial intelligence	Machine learning, deep learning, IA, DL	RQ1, RQ2, RQ3
Convolutional neural network	CNN, ConvNet	RQ1
Vision transformer	ViT, transformer	RQ1
Cervical cytology	Pap smear, cervical cells, cervical cytological images	RQ1, RQ2, RQ3
Dataset	Database, image collection, image repository	RQ2
Dataset quality	Public dataset, private dataset, balanced dataset	RQ2
Performance metrics	Accuracy, precision, recall, F1-score, AUC, sensitivity, specificity	RQ3
Model comparison	Architecture comparison, model evaluation	RQ1
Classification	Cell classification, lesion classification, lesion detection	RQ1, RQ3

### 2.4 Relevant fields for data extraction

During the data extraction phase, key metadata fields were defined to systematize the analysis of selected studies (see Table 5).

**Table 5:** Relevant data extraction fields.

**Field**	**Description**
Reference	Article title, authors, and citation.
Publication	Type of publication (journal article, conference, preprint).
Year	Year of publication.
Dataset	Name, type (public/private), size, and characteristics.
Dataset type	Public or private.
Classical dataset	Indicates whether the dataset is classical (Y/N).
Main idea	Summary of the core concept of the study.
Main contributions	Novel contributions of the article to AI in cervical cytology.
Gaps or limitations	Limitations, problems, or research gaps identified in the study.
Methodology	Methods and models used (e.g., neural networks, training techniques, hybrid architectures).
Results	Model performance outcomes (metrics).
AI models	List of AI models used (CNN, ViT, hybrid, etc.).
Model type	Indicates if the model is monolithic or hybrid.
Datasets	List of datasets used in the study.
Performance metrics	Reported metrics and their values (Accuracy, F1-score, AUC, etc.).

As part of the data extraction process, AI models were categorized according to their structural nature. Monolithic models were defined as those relying on a single deep learning architecture, while hybrid models were defined as architectures that integrate two or more complementary computational strategies within the same pipeline. Examples of hybrid approaches include CNNs combined with traditional classifiers (e.g., SVM, Random Forest, XGBoost), CNNs enhanced with fuzzy logic or evolutionary algorithms, and CNNs integrated with transformer or attention modules. This categorization allowed us to systematically compare single-architecture strategies with more complex, multi-stage approaches.

### 2.5 Information sources

The literature search was conducted in the Scopus database, including only peer-reviewed original research articles. Search strategies were independently defined for each research question (RQ1, RQ2, and RQ3) and executed in June 2025. Filters were applied based on publication year, document type (scientific articles only), and access type (Gold Open Access and Hybrid Gold). The selected records were exported in RIS format for further analysis. To avoid duplicates, cross-checking was performed between result sets from each research question. Full-text evaluation was then conducted to ensure each article met the established inclusion criteria.

The full-text evaluation results for each research question were systematically recorded in dedicated spreadsheets. These final datasets, corresponding to RQ1, RQ2, and RQ3, are available as Supplementary material in the files RQ11.xlsx, RQ21.xlsx, and RQ31.xlsx, respectively.

### 2.6 Quality appraisal

To evaluate the methodological robustness and potential risk of bias of the included studies, we implemented a structured quality appraisal adapted from the QUADAS-2 framework, which is widely used for diagnostic accuracy research.

In addition, the appraisal was structured as a checklist aligned with key criteria frequently recommended for AI studies in medical imaging: dataset transparency, validation protocol rigor, and completeness of statistical reporting.

In the context of artificial intelligence applied to cervical cytology, the appraisal focused on four key aspects: the representativeness of the datasets, the validation strategy employed the extent to which performance metrics were reported beyond accuracy, and the transparency of model description and training procedures.

Each article was assessed according to these domains, and an overall risk of bias was subsequently assigned as low, high, or unclear, based on predefined decision rules. Studies were considered low risk when they met most of the criteria satisfactorily, high risk when multiple domains were judged inadequate, and unclear when reporting was insufficient to permit confident assessment.

The per-study evaluations are available in Supplementary Table RQ31.xls, where additional columns have been included to document the quality appraisal domains and the overall risk of bias.

## 3 Results

### 3.1 Selected articles and general characteristics

Following the search strategies defined for each research question, 534 records were initially identified for RQ1, 456 for RQ2, and 381 for RQ3. Filters were subsequently applied based on publication year (2022–2025), document type (journal articles only), and access type (Gold and Hybrid Gold), which reduced the datasets to 91 articles for RQ1, 94 for RQ2, and 75 for RQ3.

A crosschecking process was then carried out to remove duplicate records across the three sets. 68 duplicates were found between RQ1 and RQ2, 57 between RQ1 and RQ3, and 54 between RQ2 and RQ3. Moreover, 47 articles were common to all three-search results. After removing duplicates, a consolidated set of 117 unique articles was obtained.

Each article was reviewed in full text to confirm its relevance. As a result, 14 articles were excluded from RQ1, 18 from RQ2, and 4 from RQ3. The main reasons for exclusion included: focus on colposcopic images, the use of non-cytological diagnostic modalities, or lack of relevant information aligned with this review's objectives. Table 6 summarizes the distribution of articles by research question and filtering stage.

**Table 6:** Distribution of articles by research question.

**Research question**	**Identified (unfiltered)**	**From 2022**	**Journal articles**	**Gold + hybrid gold**
RQ1	534	370	217	91
RQ2	456	333	208	94
RQ3	381	271	175	75

The column “Journal Articles” refers to documents that meet the required publication type (peer-reviewed journals), and the “Gold + Hybrid Gold” column shows the final set selected for analysis.

**Figure 1:**
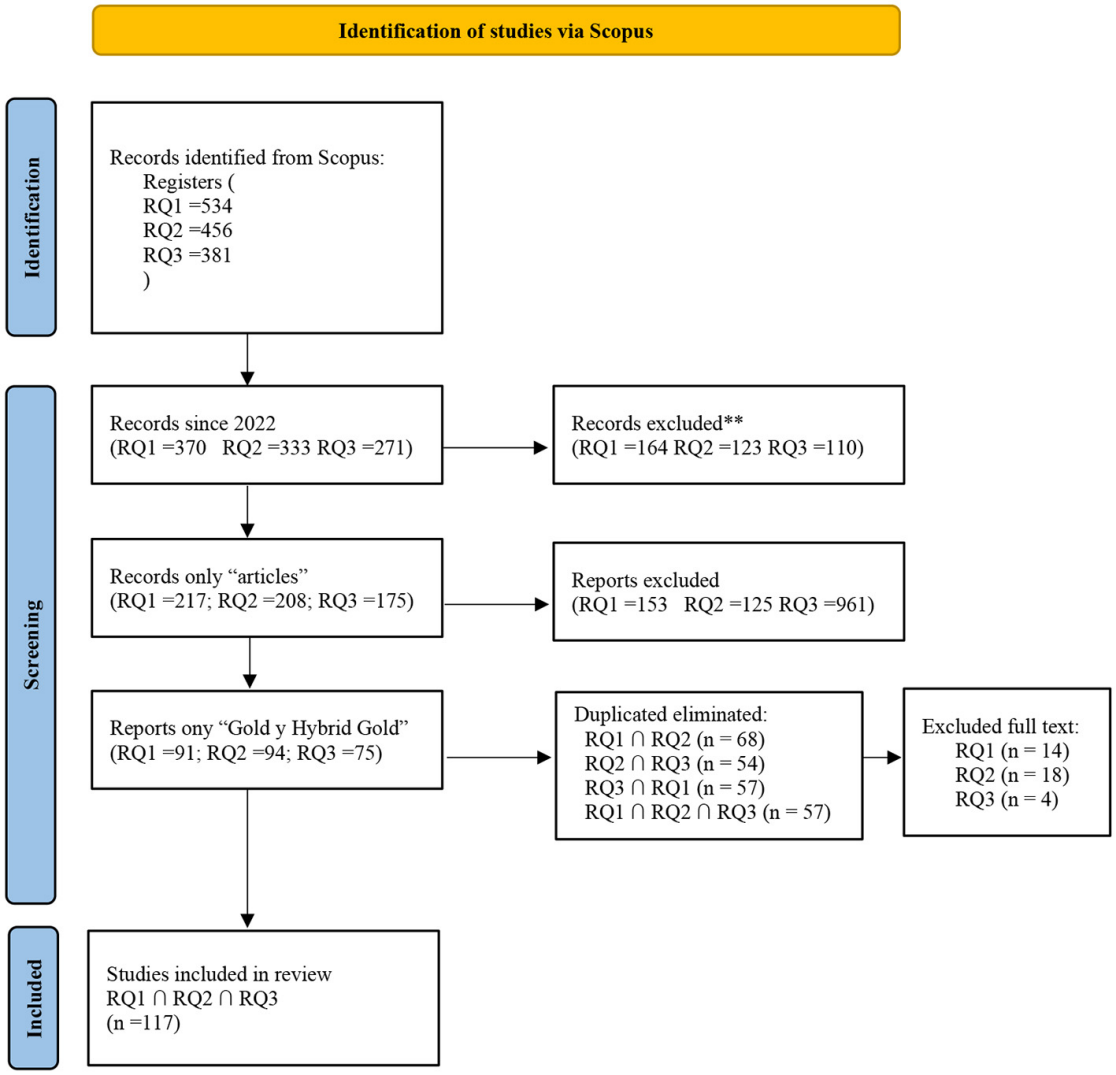
PRISMA 2020 flow diagram for study selection.

A PRISMA 2020 flow diagram was generated to illustrate the study identification, screening, and inclusion process (Figure 1).

Table 7 shows the distribution of the selected articles by year of publication. The year 2024 accounts for the highest number of articles across all three research questions, reflecting growing scientific interest in the application of AI to cervical cytology in recent years.

**Table 7:** Distribution of selected articles by year and research question (2022–2025).

**Year**	**RQ1**	**RQ2**	**RQ3**
2022	22	16	22
2023	23	24	19
2024	29	29	27
2025	4	6	3
Total	77	75	71

This annual distribution also reveals a rising trend in scientific output from 2022 to 2024 in the areas related to lesion classification, specialized dataset usage, and the evaluation of AI model performance metrics in cervical cytology imaging.

### 3.2 RQ1: artificial intelligence models applied to cervical cytology

The analysis of the 77 articles selected for RQ1 revealed a wide range of approaches for applying artificial intelligence (AI) models to the classification of cervical cytology images. Most studies implemented models based on convolutional neural networks (CNNs), followed by more recent architectures such as Vision Transformers (ViTs) and, to a lesser extent, transfer learning techniques with pretrained models. There is also growing interest in hybrid models that combine multiple techniques or stages, such as feature fusion via CNNs with traditional classifiers (e.g., SVM, XGBoost), or the integration of sequential models with attention modules.

Table 8 summarizes the frequency with which different types of models were reported in the analyzed studies. While CNNs remain prevalent, there has been a significant increase in the use of hybrid architectures over the past 3 years, suggesting a trend toward more complex and adaptive solutions.

**Table 8:** Frequency of AI models used in cervical cytology studies.

**Model type**	**Frequency**	**Percentage**	**References**
Decision trees	2	3%	Devi et al., 2023; Kalbhor et al., 2022
CNN	24	31%	Khozaimi and Mahmudy, 2024; Mazroa et al., 2023; Rasheed et al., 2023; Wong et al., 2023; Resmi et al., 2024; Attallah, 2023; Tan et al., 2024; Skerrett et al., 2022; Ahmed et al., 2024; Xu et al., 2022; Utomo et al., 2025; Shandilya et al., 2024; Priya and Bai, 2024; Zammataro, 2024; Shiny and Parasuraman, 2023; Akash et al., 2024; Wubineh et al., 2024a; Alohali et al., 2024; Zhang et al., 2025; Rodríguez et al., 2024; Tian et al., 2024; Anandavally and Bai, 2024; Kurita et al., 2023; Fang et al., 2022
Hybrid	47	61%	Tang et al., 2023; Shinde et al., 2022; Wang et al., 2024; Rohini and Kavitha, 2024; Gao et al., 2022; Song et al., 2024; Kalbhor et al., 2023c; Alquran et al., 2022a; Pang et al., 2025; Ahishakiye and Kanobe, 2024; AlMohimeed et al., 2024; Jain et al., 2024; Maurya et al., 2024; Alsalatie et al., 2022; Lilhore et al., 2022; Gangrade et al., 2025; Chauhan et al., 2023; Wang et al., 2025; Anupama et al., 2022; Mansouri and Ragab, 2023; Bora et al., 2022; Battula and Sai Chandana, 2023; Kalbhor et al., 2023a; Fekri-Ershad and Alsaffar, 2023; Battula and Chandana, 2022; AbuKhalil et al., 2022; Joynab et al., 2024; Du et al., 2023; Alquran et al., 2022b; Diniz et al., 2022; Yi et al., 2024; Chowdary et al., 2023; Chen et al., 2022; Stegmüller et al., 2024; Alsalatie et al., 2023; Benhari and Hossseini, 2022; Mahmoud et al., 2022; Ji et al., 2022; Bera et al., 2024; Ando et al., 2024; Sahoo et al., 2023; Waly et al., 2022; Akbar et al., 2024; Fahad et al., 2024; Mathivanan et al., 2024; Nour et al., 2024; Fang et al., 2024
Ensembles	3	4%	Karamti et al., 2023; Khanarsa and Kitsiranuwat, 2024; Kurniawati and Prabowo, 2022
Total	**77**	100%	

**Figure 2:**
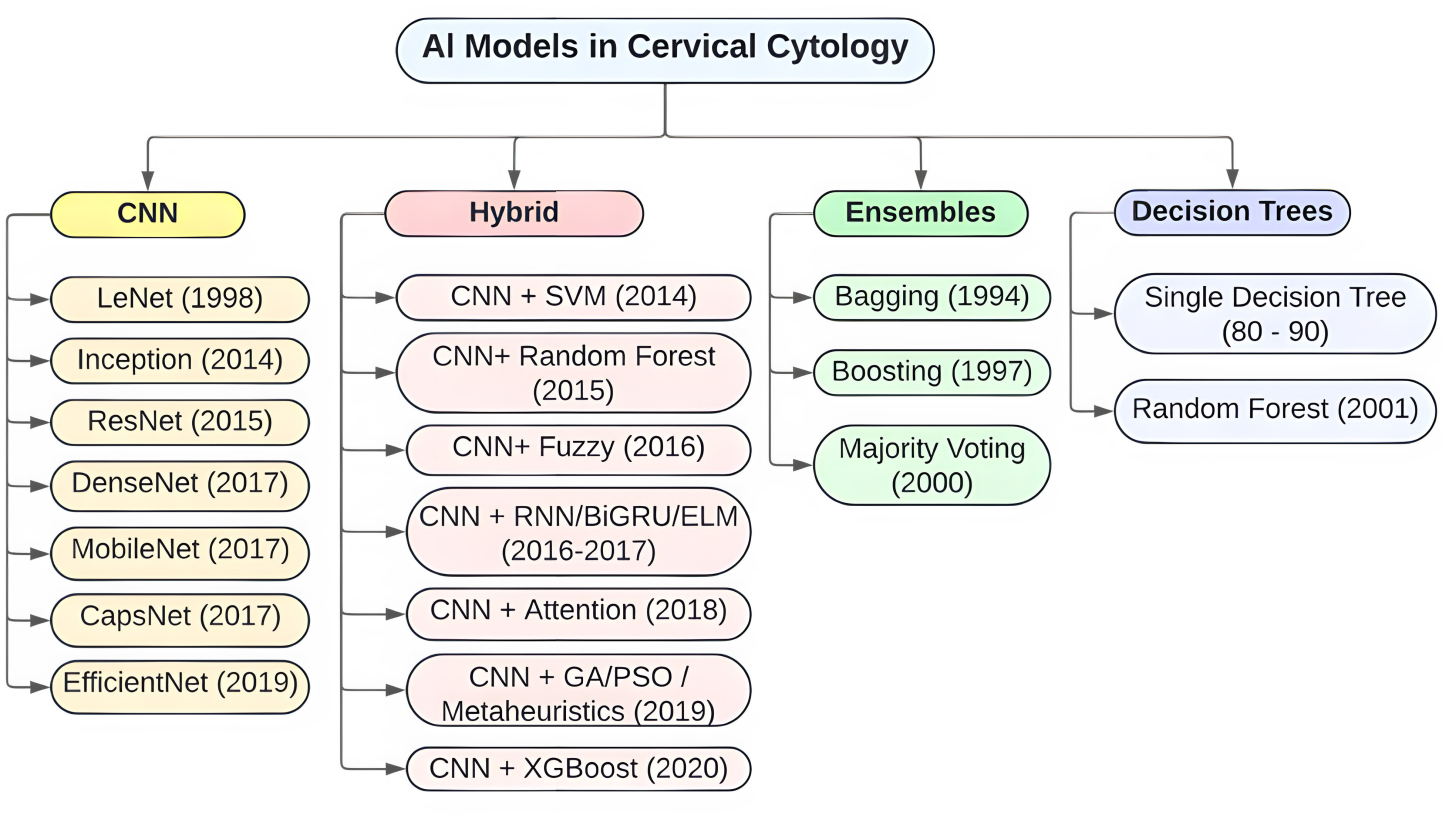
Hierarchical taxonomy of AI models reported in cervical cytology studies.

To complement the quantitative distribution shown in Table 8, Figure 2 illustrates a taxonomy of the AI models reported in the reviewed studies. This schematic representation highlights the hierarchical organization of the main categories—CNN-based models, Hybrid models, Ensembles, and Decision Trees—together with their most frequently used sub-architectures (e.g., ResNet, DenseNet, EfficientNet for CNNs, and CNN+SVM, CNN+XGBoost, CNN+Fuzzy for Hybrids). The figure provides a conceptual overview that facilitates understanding of how different computational strategies have been applied to cervical cytology, and emphasizes the predominance of hybrid approaches, which accounted for 61% of all included studies.

Additionally, the models were categorized based on their structural nature into two groups: monolithic, referring to those using a single deep learning architecture; and hybrid, defined in this review as architectures that integrate two or more complementary computational strategies within the same pipeline. Hybrid approaches included, for example, CNNs combined with traditional classifiers (e.g., SVM, Random Forest, XGBoost), CNNs enhanced with fuzzy logic or evolutionary algorithms, and CNNs integrated with transformer or attention modules. Hybrid models accounted for 61% of the reviewed articles, surpassing monolithic approaches (44%). This finding reflects a growing preference for composite strategies, which better address variability in cytological images, combine multiple feature sources, and improve classification accuracy.

In terms of temporal trends, a clear shift was observed from traditional CNN-based models to more sophisticated hybrid architectures. Between 2022 and 2024, there was an increase in the incorporation of attention modules, transformer layers, and ensemble strategies, highlighting the influence of recent advances in computer vision.

Several studies also emphasized the specific advantages of hybrid models, such as greater robustness to intercellular variability and improvements in performance metrics when combining classifiers. However, they also acknowledged limitations, including increased computational complexity, reduced reproducibility, and the need for larger annotated datasets to effectively train the additional modules.

### 3.3 RQ2: datasets used in the studies

A detailed review of the selected articles revealed 74 records in which the datasets used were explicitly stated. The findings indicate a strong reliance on classic datasets, particularly Herlev and SIPaKMeD, which were used in 16 and 15 studies, respectively. Additionally, 10 studies combined both datasets, likely to increase class diversity or improve training performance. This dominance can be attributed to their public availability, well-structured annotations, and broad dissemination within the scientific community.

In contrast, there is a growing trend toward the use of emerging or proprietary datasets. Eight studies reported the use of private datasets generated by the authors themselves, highlighting efforts to develop data contextualized to specific clinical cases or newer acquisition technologies (e.g., whole slide imaging or liquid-based cytology). Other datasets such as Mendeley LBC, ComparisonDetector, ISBI-2014/2015, and CRIC are also gaining attention for their variety of cell types and complex annotations.

Table 9 summarizes the frequency of dataset usage, with less frequently used datasets grouped under “Others.”

**Table 9:** Distribution of datasets used in AI studies for cervical cytology.

**Dataset**	**Individual Cells (A)**	**Partial Microscope Fields (B)**	**A + B**	**Frequency**	**References**
Herlev	16	0	0	16	Song et al., 2024; Resmi et al., 2024; Attallah, 2023; Tan et al., 2024; Priya and Bai, 2024; Akash et al., 2024; Anandavally and Bai, 2024; Jain et al., 2024; Anupama et al., 2022; Ando et al., 2024; Waly et al., 2022; Nour et al., 2024; Kurniawati and Prabowo, 2022; Janani and Christopher, 2023; Lotfi and Momenzadeh, 2022; Qin et al., 2022
SIPaKMeD	6	1	8	15	Tang et al., 2023; Wang et al., 2024; Chauhan et al., 2023; Khozaimi and Mahmudy, 2024; Gao et al., 2022; Utomo et al., 2025; Shandilya et al., 2024; Wubineh et al., 2024a; Alohali et al., 2024; Kurita et al., 2023; Maurya et al., 2024; Gangrade et al., 2025; Fekri-Ershad and Alsaffar, 2023; Ontor et al., 2023; Mathivanan et al., 2024
SIPaKMeD + Herlev	0	2	8	10	Shinde et al., 2022; Kalbhor et al., 2023b; Zhang et al., 2025; Fang et al., 2022; AlMohimeed et al., 2024; Chowdary et al., 2023; Benhari and Hossseini, 2022; Chen et al., 2022; Zhao et al., 2023; Fahad et al., 2024
Private	2	1	5	8	Bora et al., 2022; Joynab et al., 2024; Yi et al., 2024; Stegmüller et al., 2024; Liu et al., 2022; Riana et al., 2023; Yin et al., 2024; An et al., 2023
Mendeley LBC	3	0	2	5	Wong et al., 2023; Shiny and Parasuraman, 2023; Rodríguez et al., 2024; Battula and Chandana, 2022; Sahoo et al., 2023
ISBI-2014/2015	2	1	2	5	Zhang et al., 2024a; Mahyari and Dansereau, 2022; Zhang et al., 2024b; Kalbhor et al., 2023a; Rasheed et al., 2023
Comparison Detector	0	1	2	3	Pang et al., 2025; Cheng et al., 2025; Li et al., 2025
SIPaKMeD + CRIC	0	0	2	2	Battula and Sai Chandana, 2023; Fang et al., 2024
Others (11 datasets)	3	2	5	10	Kang and Li, 2024; Xu et al., 2022; Kalbhor et al., 2023c; Yang et al., 2022; Galande et al., 2024; Albuquerque et al., 2023; Ji et al., 2023; Nazir et al., 2023; Luo et al., 2022; Wu et al., 2023a
Total	13	6	45	74	

**Figure 3:**
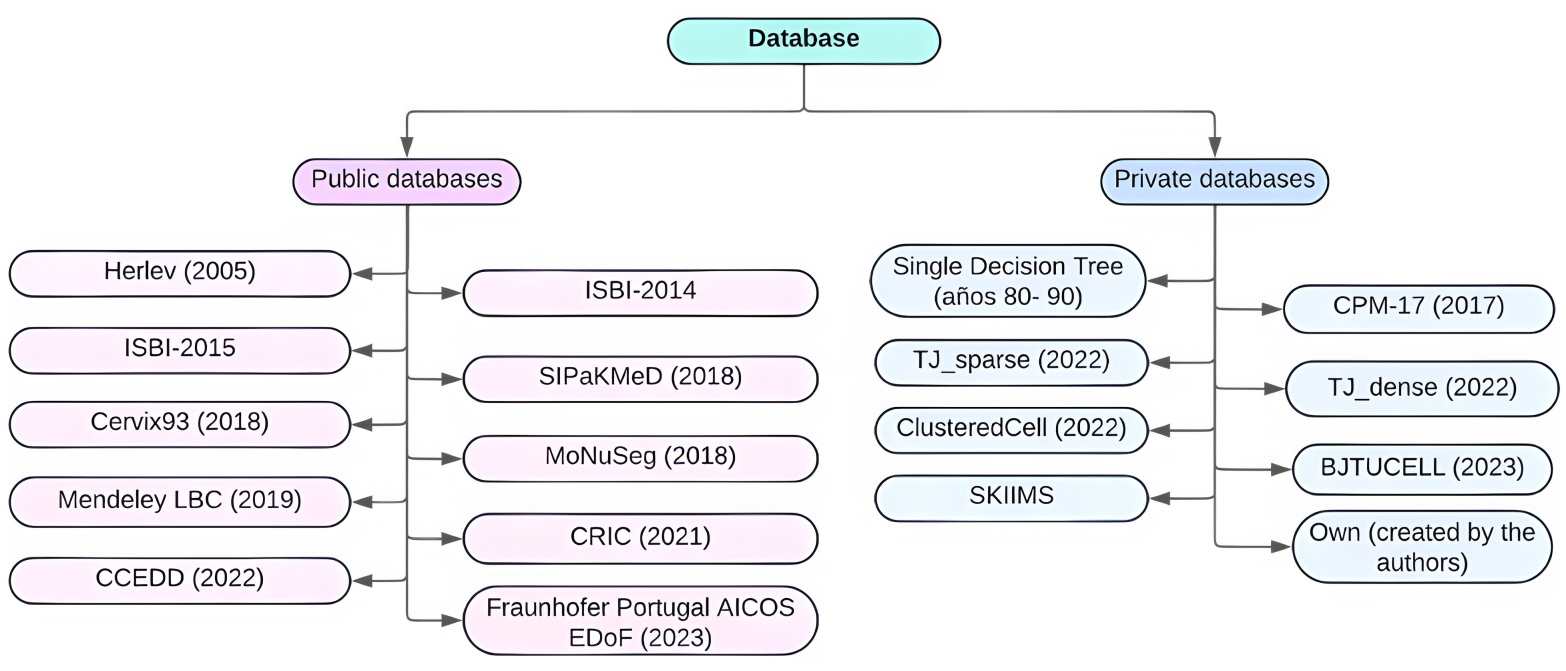
Dataset taxonomy in cervical cytology AI research: source type, dataset name, and image modality.

To complement the descriptive distribution presented in Table 9, Figure 3 illustrates a hierarchical taxonomy of datasets applied in cervical cytology studies. The classification begins by distinguishing between public and private datasets, then specifies the individual datasets most frequently reported (e.g., Herlev, SIPaKMeD, Mendeley LBC, ISBI-2014/2015, and others), and finally maps the type of image analyzed in each case (individual cells, partial microscope fields, or combined approaches). This visualization highlights the dominance of public datasets, particularly Herlev and SIPaKMeD, but also reveals an emerging contribution of private institutional collections that integrate partial fields or whole-slide derivatives. Such a taxonomy not only clarifies the methodological landscape but also underscores the heterogeneity in data sources and image modalities, which directly affects the comparability and generalizability of AI models in cervical cytology.

Regarding dataset type, the analysis reveals that **69%** of the studies relied on **public datasets**, while **31%** used **private or self-generated datasets**. This distribution underscores the importance of open data in scientific reproducibility, while also emphasizing the need to expand the diversity—both demographic and technological—of training sets.

In terms of image types, three major approaches were identified: **Individual Cell Images (A)** – most common. **Partial Microscopy Fields (B)** – derived from WSI (whole slide images), capturing spatial and contextual features. **Combined A** **+** **B** – integrating both approaches. Although the use of tiles from WSIs remains emerging, it is seen as a growing trend, especially with the advent of models that are more sophisticated and the need for clinical scalability.

While public datasets enhance comparability across studies, they also present risks of overfitting, limited class diversity and poor representativeness of morphological variants from different populations. On the other hand, private datasets face challenges in access and validation but offer opportunities for personalized diagnostic solutions tailored to real-world clinical settings.

### 3.4 RQ3: performance metrics and results obtained

Although most studies reported high accuracy values, the quality assessment revealed frequent methodological limitations. The main issues included overreliance on classical public datasets, lack of external validation, incomplete reporting of class-level metrics, and insufficient description of training procedures. These patterns suggest that the reported performance must be interpreted with caution. Detailed assessments for each study are provided in the Supplementary material (see Supplementary Table RQ31.xls, with extended fields for quality appraisal).

The analysis of the studies included in this systematic review reveals a predominant use of traditional classification metrics to evaluate the performance of artificial intelligence (AI) models applied to cervical cytology images. The most frequently reported metrics were accuracy, precision, recall, F1-score, specificity, and area under the ROC curve (AUC). This selection reflects a focus not only on overall classification accuracy but also on the models' ability to detect minority classes, which is critical in clinical contexts.

Among the 71 reviewed articles, 93.9% reported accuracy as their primary metric, both in binary and multiclass classification schemes. Accuracy values ranged from 63.08 to 100%, with the highest performances associated with Vision Transformer (ViT)-based models and hybrid architectures. Quantitative analysis yielded a mean accuracy of 87.76%, making it the recurrent metric across the 121 recorded performance entries.

Precision and recall were reported in approximately 65% of the studies, highlighting growing attention to class-level performance and the trade-off between true positives and false negatives. The mean precision was 87.01%, while recall averaged 78.06%, with wide variation across models, suggesting differences in how class imbalance was handled. The F1-score, used in 54% of the studies, had an average of 64.65%, but reached values close to 99% in well-optimized multiclass models, especially those evaluated on datasets such as SIPaKMeD.

Table 10 presents a statistical summary of the most frequently reported performance metrics, including frequency, mean, and observed minimum and maximum values, along with references to the studies that used them as primary metrics.

**Table 10:** Frequency and statistical values of performance metrics reported in the reviewed studies.

**Metric**	**Frequency**	**Avg (%)**	**Min (%)**	**Max (%)**	**References**
Accuracy	64	94.95	58.00	100.0	Tang et al., 2023; Rohini and Kavitha, 2024; Khozaimi and Mahmudy, 2024; Gao et al., 2022; Song et al., 2024; Kalbhor et al., 2022; Wong et al., 2023; Attallah, 2023; Tan et al., 2024; Ahmed et al., 2024; Shandilya et al., 2024; Priya and Bai, 2024; Zammataro, 2024; Shiny and Parasuraman, 2023; Akash et al., 2024; Wubineh et al., 2024a; Zhang et al., 2025; Rodríguez et al., 2024; Tian et al., 2024; Anandavally and Bai, 2024; Kurita et al., 2023; Kalbhor et al., 2023c; Alquran et al., 2022a; Pang et al., 2025; Ahishakiye and Kanobe, 2024; AlMohimeed et al., 2024; Jain et al., 2024; Maurya et al., 2024; Alsalatie et al., 2022; Gangrade et al., 2025; Chauhan et al., 2023; Wang et al., 2025; Bora et al., 2022; Battula and Sai Chandana, 2023; Kalbhor et al., 2023a; Fekri-Ershad and Alsaffar, 2023; Battula and Chandana, 2022; AbuKhalil et al., 2022; Joynab et al., 2024; Alquran et al., 2022b; Diniz et al., 2022; Yi et al., 2024; Alsalatie et al., 2023; Benhari and Hossseini, 2022; Sahoo et al., 2023; Waly et al., 2022; Akbar et al., 2024; Karamti et al., 2023; Khanarsa and Kitsiranuwat, 2024; Kurniawati and Prabowo, 2022; Lotfi and Momenzadeh, 2022; Mathivanan et al., 2024; Chen et al., 2022; Fahad et al., 2024; Alohali et al., 2024; Sudhakar et al., 2023; Petrov and Sokolov, 2023; Lilhore et al., 2022; Yang et al., 2024; Harsono et al., 2022; Haridas and Jayamalar, 2023; Wubineh et al., 2024b; Shinde et al., 2022
Precision	25	92.61	60.78	100.0	Tang et al., 2023; Chowdary et al., 2023; Wong et al., 2023; Priya and Bai, 2024; Shiny and Parasuraman, 2023; Tian et al., 2024; Anandavally and Bai, 2024; Fang et al., 2022; Ahishakiye and Kanobe, 2024; AlMohimeed et al., 2024; Battula and Sai Chandana, 2023; AbuKhalil et al., 2022; Diniz et al., 2022; Mahmoud et al., 2022; Waly et al., 2022; Karamti et al., 2023; Kurniawati and Prabowo, 2022; Chen et al., 2022; Kalbhor et al., 2023a; Luo et al., 2022; Alohali et al., 2024; Sudhakar et al., 2023; Harsono et al., 2022; Haridas and Jayamalar, 2023; Shinde et al., 2022
Recall	35	93.46	66.10	100.0	Tang et al., 2023; Chowdary et al., 2023; Gao et al., 2022; Song et al., 2024; Attallah, 2023; Xu et al., 2022; Priya and Bai, 2024; Shiny and Parasuraman, 2023; Rodríguez et al., 2024; Tian et al., 2024; Anandavally and Bai, 2024; Fang et al., 2022; Ahishakiye and Kanobe, 2024; AlMohimeed et al., 2024; Maurya et al., 2024; Battula and Sai Chandana, 2023; Battula and Chandana, 2022; AbuKhalil et al., 2022; Diniz et al., 2022; Benhari and Hossseini, 2022; Mahmoud et al., 2022; Sahoo et al., 2023; Waly et al., 2022; Karamti et al., 2023; Kurniawati and Prabowo, 2022; Chen et al., 2022; Kalbhor et al., 2023a; Luo et al., 2022; Alohali et al., 2024; Sudhakar et al., 2023; Petrov and Sokolov, 2023; Lilhore et al., 2022; Harsono et al., 2022; Haridas and Jayamalar, 2023; Shinde et al., 2022
Specificity	14	89.40	48.80	99.09	Chowdary et al., 2023; Gao et al., 2022; Song et al., 2024; Rodríguez et al., 2024; Maurya et al., 2024; Battula and Chandana, 2022; Benhari and Hossseini, 2022; Ando et al., 2024; Chen et al., 2022; Sudhakar et al., 2023; Petrov and Sokolov, 2023; Harsono et al., 2022; Haridas and Jayamalar, 2023; Wubineh et al., 2024b
F1-score	23	94.14	62.82	100.0	Tang et al., 2023; Song et al., 2024; Attallah, 2023; Priya and Bai, 2024; Shiny and Parasuraman, 2023; Tian et al., 2024; Anandavally and Bai, 2024; Fang et al., 2022; Ahishakiye and Kanobe, 2024; AlMohimeed et al., 2024; Maurya et al., 2024; Battula and Sai Chandana, 2023; AbuKhalil et al., 2022; Diniz et al., 2022; Mahmoud et al., 2022; Sahoo et al., 2023; Waly et al., 2022; Karamti et al., 2023; Kurniawati and Prabowo, 2022; Kalbhor et al., 2023a; Lilhore et al., 2022; Haridas and Jayamalar, 2023; Shinde et al., 2022

Specificity was reported less frequently (15%), typically in models that incorporated probabilistic outputs or clinical attention modules. It was more common in studies designed to simulate real-world medical validation. Metrics such as balanced accuracy, Matthews's correlation coefficient, and AUC were less common, but their appearance has increased in studies published since 2023—an indication of evolving practices toward more clinically meaningful and balanced evaluations.

From a comparative perspective, hybrid models (e.g., CNN–ViT combinations or architectures with attention mechanisms) achieved the highest average accuracy (96.63%), followed by CNN-based models (e.g., ResNet, DenseNet) with an average of 94.91%. In contrast, ensemble and classical models as if Random Forest exhibited lower performance, with average accuracy around 63–83%, depending on the dataset used (Table 11).

**Table 11:** Comparison of metrics by architecture type.

**Metric**	**Accuracy**			**Precision**			**Recall**			**F1 Score**		
	**Min**	**Avg**	**Max**	**Min**	**Avg**	**Max**	**Min**	**Avg**	**Max**	**Min**	**Avg**	**Max**
CNN	72.80	94.91	100.00	80.00	92.06	99.00	77.40	93.36	100.00	82.50	92.98	100.00
Ensemble	63.08	63.08	63.08	60.78	60.78	60.78	66.10	66.10	66.10	62.82	62.82	62.82
Hybrid	81.11	96.63	100.00	68.00	95.15	100.00	70.00	95.46	100.00	89.00	96.61	100.00
VIT	99.11	99.11	99.11	99.12	99.12	99.12	99.11	99.11	99.11	99.11	99.11	99.11

In summary, the metrics reported reveal a favorable outlook for AI-based models in cervical cytology, with performance levels that match or even exceed human-level diagnosis in specific tasks. Nonetheless, common limitations persist, including inconsistent result reporting, lack of external cross-validation, and limited discussion of the statistical significance of differences between models. These aspects must be addressed in future research to ensure reliable, clinically robust, and ethically sound AI implementations.

### 3.5 Cross-analysis: relationships between models, datasets, and metrics

The cross-analysis of model types, datasets used, and reported performance metrics reveals emerging patterns and significant associations that characterize the current development of AI-based models in cervical cytology.

A predominance of convolutional neural networks (CNNs) and hybrid architectures (e.g., CNN + Transformer, CNN + RNN, or attention-enhanced models) was observed. These models were most frequently applied to classic datasets such as SIPaKMeD and Herlev. CNNs trained on SIPaKMeD achieved an average accuracy of 99.12%, while on Herlev the average dropped to 87.44%. This suggests a higher affinity between CNN architectures and the visual characteristics of SIPaKMeD, possibly due to its well-defined class structure and standardized preprocessing.

Hybrid models also achieved high average performance-−97.31% on SIPaKMeD and 95.30% on Mendeley LBC—surpassing pure CNNs and demonstrating their ability to capture complex morphological relationships. Notably, in more heterogeneous datasets like Cervix93, which include greater variability and less uniformity in the samples, hybrid models still maintained high accuracy levels (up to 99.01%), highlighting their robustness.

In contrast, decision tree models and those based on traditional machine learning techniques showed lower average performance (83.00% in mixed datasets) and appeared less frequently in recent studies, likely due to their limitations when dealing with complex, multiclass cytological images.

There is a clear tendency to report better metrics when using classic datasets like SIPaKMeD and Herlev. These datasets are not only widely used, but also offer more consistency in terms of resolution, annotation, and class balance—factors that favor the training and evaluation of deep learning models. However, this dependency on classic datasets poses a significant limitation for clinical generalization, as they do not capture the full variability of real-world cytological environments.

On the other hand, models trained on private or non-traditional datasets have shown competitive metrics, but the lack of public availability and inconsistent annotation standards hinder direct comparison and limit the reproducibility of results.

This analysis indicates that although hybrid architectures offer superior performance in controlled scenarios, there remains an overreliance on a small set of classic datasets. Future research must prioritize evaluating these models in real clinical settings, incorporating whole-slide images (WSI) and multisource data. Additionally, there is a need to develop standardized multiclass and multimodal benchmarks, and to encourage the open publication of expert-annotated datasets.

Furthermore, the research community should move toward the systematic use of complementary metrics (e.g., balanced accuracy, negative predictive value, Kappa coefficient) and ensure external cross-validation and the reporting of confidence intervals. These practices are essential to promote transparency, reproducibility, and clinical applicability of the proposed AI models.

The encouraging results of AI models in cervical cytology should be considered in light of their methodological limitations. Our quality appraisal revealed frequent risks of bias, including reliance on small or homogeneous datasets, absence of external validation, and incomplete reporting of clinically relevant metrics. These issues may inflate reported performance values and limit generalizability. Future research should prioritize representative datasets, standardized reporting frameworks, and external validation to ensure robust and clinically reliable evidence.

## 4 Discussion

### 4.1 AI Models: from CNN predominance to hybrid strategies

The most striking finding of this review is the shift from CNN dominance toward hybrid architectures and, more recently, Vision Transformers (AlMohimeed et al., 2024; Yamagishi and Hanaoka, 2025; Muksimova et al., 2024). This is not a trivial transition: CNNs have demonstrated robustness but also clear limitations in capturing global dependencies within cytology images, as also noted by Muksimova et al. (2025) and Mustafa et al. (2025). The fact that more than 60% of recent studies rely on hybrid combinations shows how the field is trying to address morphological variability (Table 8). However, this increasing sophistication comes with trade-offs: while hybrid models can boost metrics, they often do so at the cost of reproducibility, transparency, and computational feasibility in low-resource settings. The tension between technical precision and clinical applicability remains unresolved in the literature.

### 4.2 Datasets: the paradox of public versus private

Regarding datasets, the field still depends heavily on SIPaKMeD and Herlev. These collections are valuable as benchmarks, but their overuse introduces an evident bias: they fail to represent the population diversity and preparation variability encountered in real-world practice (Ybaseta-Medina et al., 2025). It is telling that even the most sophisticated models can reach near-perfect accuracy on these “clean” datasets, while performance drops when evaluated on more heterogeneous data (Pang et al., 2025; Joynab et al., 2024; Wu et al., 2023a; Khan et al., 2023; Wu et al., 2023b). Attempts to develop private or institutional datasets are commendable because they move closer to clinical contexts, but their lack of public availability prevents replication and fair comparison. This gap seriously undermines the community's ability to establish robust standards.

### 4.3 Metrics and evaluation practices: beyond accuracy

Although accuracy remains the most frequently reported metric (Table 10), this emphasis is problematic. Global accuracy can inflate perceptions of success while masking poor performance in minority but clinically critical classes, such as HSIL or SCC (Ando et al., 2024; Cheng et al., 2025; Suksmono et al., 2021). The fact that fewer than 20% of studies reported metrics such as specificity, negative predictive value, or balanced accuracy reflects insufficient maturity in evaluation design. This shortfall is not merely technical: it has direct consequences for patient safety, as a model that maximizes accuracy at the expense of sensitivity in HSIL cannot be trusted in clinical decision-making. Future research must therefore standardize validation protocols, incorporate external validation, and adopt metrics that capture the real clinical cost of misclassification.

### 4.4 Cross-analysis: patterns and warnings

The cross-analysis of models, datasets, and metrics uncovers a paradox that cannot be overlooked: the best results cluster around classical datasets with relatively simple structures, while more realistic scenarios — whole-slide images and heterogeneous institutional collections — remain underexplored, as also noted in the review by Jiang et al. (2023). This reveals a persistent gap between academic research and clinical application. Moving forward, the field must prioritize multicenter benchmarks with diverse data and more rigorous evaluation criteria. Only then, can AI in cervical cytology move beyond being a promising academic exercise and evolve into a clinically reliable and ethically sound tool.

## 5 Conclusions

This systematic review provides an integrative perspective on the application of artificial intelligence in cervical cytology, focusing on deep learning models, datasets, and performance outcomes. Through the analysis of 77 peer-reviewed articles published between 2022 and 2025, we identified a clear predominance of convolutional neural networks and hybrid architectures—particularly those combining CNNs with attention mechanisms or transformer-based models—as the core computational strategies for lesion classification.

In terms of data usage, the review revealed a significant dependency on a small number of publicly available datasets, particularly SIPaKMeD and Herlev. While these datasets offer consistency and facilitate benchmarking across studies, their limited clinical variability poses a challenge for real-world generalizability. The emergence of private or custom datasets represents an important effort to diversify data sources, although lack of accessibility and annotation standards hinders replication and external validation.

Regarding model performance, most studies reported high levels of accuracy, precision, and recall, especially those employing hybrid models trained on curated datasets. However, inconsistent reporting practices, limited use of external cross-validation, and underutilization of clinically meaningful metrics such as specificity, balanced accuracy, and AUC indicate the need for more robust evaluation protocols.

To our knowledge, this is the first systematic review to conduct a cross-sectional analysis that jointly examines the relationships between deep learning architectures, dataset types, and diagnostic metrics in the context of cervical cytology. This integrative approach offers a broader understanding of current practices and challenges in the field, contributing valuable insights that may inform the development of more reliable, interpretable, and clinically aligned AI systems for early detection of cervical lesions.

In summary, the most relevant outcomes of this review are threefold: (i) the predominance of hybrid architectures, particularly CNNs combined with transformer or attention modules, as the emerging computational trend; (ii) the continued dependence on a small set of classical datasets (SIPaKMeD and Herlev), despite increasing interest in private and heterogeneous collections; and (iii) the overall performance patterns, with accuracies typically ranging between 87 and 95% and F1-scores between 64 and 96%, which underscore both the potential and the methodological limitations of current models. These findings provide a concrete reference point for future research and development of clinically applicable AI systems in cervical cytology.

## Data Availability

The original contributions presented in the study are included in the article/Supplementary material, further inquiries can be directed to the corresponding author.
